# Role of Furfural and 5-Methyl-2-furfural in Glucose-Induced Inhibition of 2-Amino-1-methyl-6-phenylimidazo[4,5-b]pyridine (PhIP) Formation in Chemical Models and Pork Patties

**DOI:** 10.3390/molecules30061254

**Published:** 2025-03-11

**Authors:** Yuexia Qin, Zhuyu Zheng, Di Liu, Shuhua Sun, Xiaolei Zhao, Lei Lv, Dengyu Xie, Zhonghui Han, Jinxing He

**Affiliations:** 1 Shandong Key Laboratory of Healthy Food Resources Exploration and Creation, College of Food Science and Engineering, Qilu University of Technology (Shandong Academy of Sciences), Jinan 250353, China; 10431221122@stu.qlu.edu.cn (Y.Q.); 202395093001@stu.qlu.edu.cn (Z.Z.); liudi92304@163.com (D.L.); zhaoxiaolei@qlu.edu.cn (X.Z.); lvlei831005@qlu.edu.cn (L.L.); 2 Shandong Engineering Research Center of Food Nutrition and Active Health, Binzhou Key Laboratory of Corn Deep Processing Technology, Shandong Xiwang Foodstuffs Co., Ltd., Binzhou 256200, China; sunshuhua7677@126.com (S.S.); dengnet@126.com (D.X.)

**Keywords:** 2-amino-1-methyl-6-phenylimidazo[4,5-b]pyridine (PhIP), furfural, 5-methyl-2-furfural, inhibition

## Abstract

The effects of furfural and 5-methyl-2-furfural produced by the Maillard reaction on PhIP formation were investigated in chemical models and roasted pork patties. In the chemical models, the results indicated that increasing levels of furfural (r = −0.7338, R^2^ = 0.9557) and 5-methyl-2-furfural (r = −0.7959, R^2^ = 0.9864) significantly reduced PhIP formation, displaying a strong linear correlation. The effects of furfural and 5-methyl-2-furfural on the precursors of phenylalanine (Phe) and phenylacetaldehyde showed a significant reduction in the Phe level, while the level of phenylacetaldehyde was not increased. In addition, neither furfural nor 5-methyl-2-furfural could significantly reduce creatinine or PhIP. Further mechanism studies showed that furfural (5-methyl-2-furfural) directly captured Phe to form the corresponding Schiff base compounds a (2-((furan-2-ylmethylene) amino)-3-phenylpropanoic acid) and b (2-(((5-methylfuran-2-yl)methylene)amino)-3-phenylpropanoic acid). This process reduced the production of phenylacetaldehyde, thereby inhibiting the PhIP formation pathway. More importantly, these two compounds were detected in roasted pork patties to which glucose was added. The above pathway was finally confirmed in roasted pork patties. These results revealed that furfural and 5-methyl-2-furfural, formed during the Maillard reaction, play a significant role in inhibiting the formation of PhIP by reacting with Phe.

## 1. Introduction

Thermal processing can enhance food’s nutritional quality, improve its nutritional digestibility, and prolong its shelf life [1]. However, some unhealthy compounds, such as heterocyclic amines (HCAs), also form during thermal processing [2]. HCAs are a class of compounds characterized by polycyclic aromatic structures that are formed during the thermal processing of protein-rich foods [3,4]. They are widely found in thermally processed meat products such as pork [5,6], beef [7,8], fish [9,10] and chicken [7,9,11]. Animal experiments and epidemiological studies have shown that HCAs increase the risk of colon cancer [12,13,14], pancreatic cancer [4,15], gastric cancer [15,16], esophageal cancer [12,15], and prostate cancer [17,18]. So far, more than 30 kinds of HCAs have been isolated and identified from food, divided into polar and non-polar categories [19,20]. Studies have found that polar HCAs are more harmful to humans [21]. The International Agency for Research on Cancer classified 2-amino-3-methylimidazo[4,5-f]quinoline (IQ) as a class 2A carcinogen and 2-amino-1-methyl-6-phenylimidazo[4,5-b]pyridine (PhIP), 2-amino-3,4-dimethylimidazo[4,5-f]quinoline (MeIQ), 2-amino-3,8-dimethylimidazo[4,5-f]quinoline (MeIQx), and so on as class 2B carcinogens in 2020 [22]. Although these HCAs are present at low levels in food, they can commonly be formed during the application of popular cooking methods. Therefore, it is significant to study the formation of HCAs in meat products to reduce the health risks of dietary exposure.

PhIP is one of the more abundant HCAs and is often used as a representative in the study of HCAs [3,23]. Studies have shown that phenylalanine (Phe) and creatinine are necessary precursors for the formation of PhIP, with Phe being involved in the formation of PhIP after Strecker degradation to form the important intermediates phenylacetaldehyde [24] and benzaldehyde [25]. Reducing sugars differ from other compounds that affect the formation of PhIP, mainly because they are not only important but are also a widely used food additive. More importantly, they are a critical component of the Maillard reaction in food. Researchers have confirmed that different types of reducing sugars can inhibit the formation of PhIP in food. For example, some researchers have found a significant correlation between HCAs in pork and glucose [5,26]. The content of PhIP in roast chicken is reduced by 77% when pickled with pickle juice containing 10% glucose [27]. Zhang et al. also found that low proportions of green tea leaves and sucrose were suitable for sensory evaluation and influenced the formation of HCAs in smoked chicken [28]. Additionally, the addition of 5% honey can reduce the content of PhIP in fried beef tenderloin and chicken breast by 45–67% [8]. In a review by Monika Gibis, it was written that glucose plays an important role in the formation of heterocyclic amines, and the formation of Maillard reaction products through reduction may provide a new approach to reducing heterocyclic amines [29]. Accordingly, studying the relationship between glucose and PhIP formation is essential for controlling PhIP in food.

Regarding the mechanisms and pathways of the glucose effect on PhIP formation, some researchers believe that the increased amount of reducing sugar leads to an increased reaction with creatinine, reducing the amount of creatinine needed for PhIP formation and ultimately resulting in decreased PhIP formation [28]. Others have suggested in reviews that this is related to the increased content of reactive carbonyl compounds in the entire system due to the addition of glucose [29]. However, most reports on the inhibition mechanism are based on theoretical analysis and speculation, which have not been effectively confirmed. Previously, Skog et al. [30,31] showed that HMF, a typical Maillard reaction product, can inhibit the formation of mutagenic activity by consuming creatinine in a model system. In addition, we found that α-dicarbonyl compounds play a complex and multifaceted role in the influence of glucose on PhIP formation and mitigation [32]. However, this does not fully elucidate the mechanism by which glucose affects PhIP formation, primarily because the Maillard reaction involving glucose is highly complex, leading to the formation of not only α-dicarbonyl compounds but also other critical reactive products such as furfural and 5-methyl-2-furfural. After our preliminary experiments, we found that furfural and 5-methyl-2-furfural were produced with added glucose in the chemical model (Figure S1). However, the role of furfural and 5-methyl-2-furfural formed by the Maillard reaction in the effect of glucose on PhIP formation is unclear.

Hence, the objectives of this study were to further elucidate the effect of the Maillard reaction involving glucose on the formation of PhIP and its underlying mechanisms through the pathways of the effects of furfural and 5-methyl-2-furfural on PhIP formation in chemical model systems and roasted pork patties. The findings of this research further enrich the understanding of the mechanism of influence of the Maillard reaction on the formation of PhIP during thermal processing and provide a theoretical basis for improving the quality of thermally processed meat products.

## 2. Results and Discussion

The addition of glucose can greatly reduce the amount of PhIP in roasted pork. During thermal processing, glucose can be easily degraded into furfural and 5-methyl-2-furfural by the Maillard reaction. To understand the effect of glucose on the formation of PhIP, further studies were performed on the effects of furfural and 5-methyl-2-furfural formed by the Maillard reaction on the formation of PhIP.

### 2.1. Effects of Furfural and 5-Methyl-2-furfural on the Formation of PhIP in the Chemical Models

**Figure 1:**
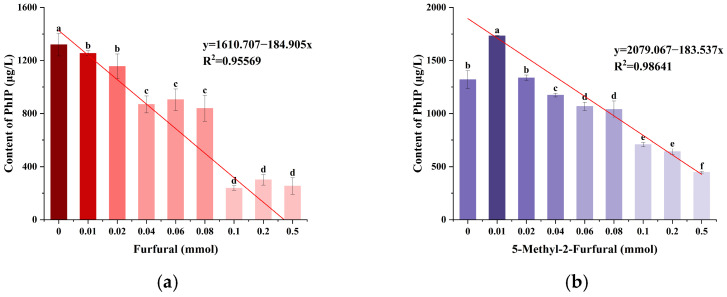
Effects of furfural (**a**) and 5-methyl-2-fural (**b**) on the formation of PhIP in the chemical models. Different lowercase letters indicate significant difference (*p* < 0.05).

In the model system, the effects of different concentrations of furfural and 5-methyl-2-furfural on PhIP formation were studied using LC-MS/MS. As shown in Figure 1a, with the increase in furfural concentration, the formation of PhIP gradually decreased (*p* < 0.05). When the furfural addition was 0.1 mmol, the inhibition rate of PhIP reached 81.94% (Table S1), and there was a strong negative correlation between the addition amount of furfural and the formation of PhIP (Pearson’s r = -0.7338, R^2^ = 0.9557). The data showed that the effect of 5-methyl-2-furfural on PhIP formation in the range of 0.02–0.5 mmol was similar to that of furfural (Figure 1b). There was also a strong negative correlation between 5-methyl-2-furfural addition and PhIP formation (Pearson’s r = −0.7959, R^2^ = 0.9864). These results indicate that furfural and 5-methyl-2-furfural significantly inhibit PhIP formation.

### 2.2. Effects of Furfural and 5-Methyl-2-Furfural on PhIP and Its Precursors in the Chemical Models

**Figure 2:**
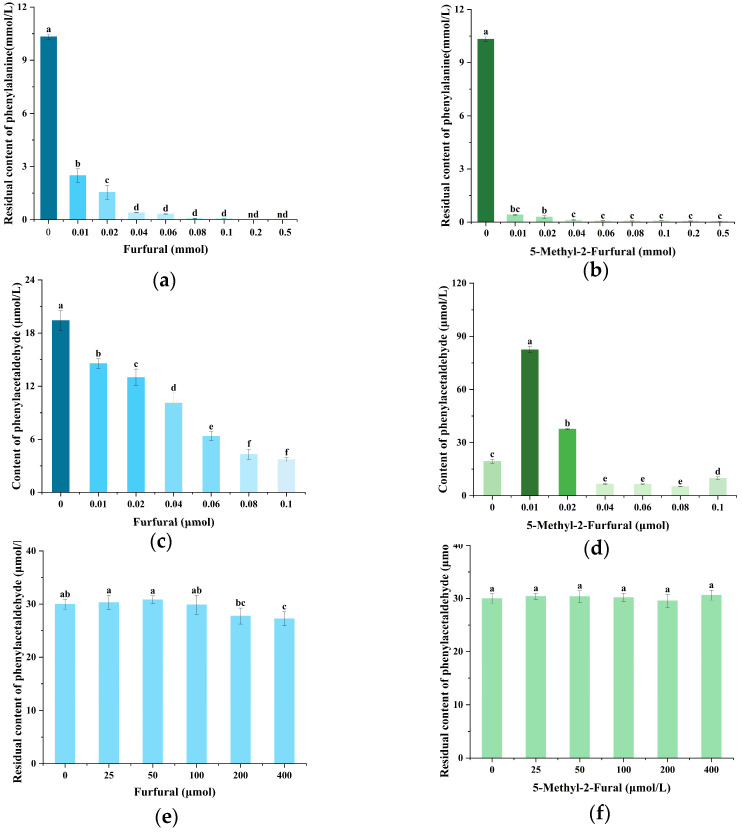
Effect of furfural (5-methyl-2-furfural) on phenylalanine (Phe) in the chemical models (**a**,**b**); effect of furfural (5-methyl-2-furfural) on phenylacetaldehyde in the chemical models of phenylacetaldehyde production by Phe (**c**,**d**); the change in phenylacetaldehyde in the chemical models of furfural (5-methyl-2-furfural) reaction with phenylacetaldehyde (**e**,**f**). Different lowercase letters indicate significant difference (*p* < 0.05).

Previous studies have shown that Phe and creatinine are the key precursors for the formation of PhIP. Phe undergoes Strecker degradation at high temperatures, converting to phenylacetaldehyde, which then reacts with creatinine to form PhIP [24]. Building on this foundation, we investigated the effect of furfural and 5-methyl-2-furfural on PhIP formation by examining the levels of Phe, creatinine, and phenylacetaldehyde in furfural/Phe (creatinine, and 5-methyl-2-furfural/Phe (creatinine) model systems. As shown in Figure 2a,b, the data demonstrated that the Phe levels decreased gradually with the increase in furfural (5-methyl-2-furfural) concentration. At a concentration of 0.01 mmol, furfural and 5-methyl-2-furfural reduce Phe by 75.84% and 95.98%, respectively. Compared with furfural, 5-methyl-2-furfural reduced Phe faster and consumed more. The above results indicated that furfural (5-methyl-2-furfural) significantly reduced the amount of Phe, with 5-methyl-2-furfural being more likely to react with Phe. Phenylacetaldehyde in the model was further studied. It can be observed in Figure 2c that the addition of furfural led to a decrease in the amount of phenylacetaldehyde generated by Phe, and the amount of phenylacetaldehyde generated gradually decreased with the increase in furfural concentration. As shown in Figure 2d, the amount of phenylacetaldehyde was significantly reduced when 5-methyl-2-furfural was added in an amount of more than 0.02 mmol. The data indicated that furfural had an inhibitory effect on the formation of phenylacetaldehyde, and 5-methyl-2-furfural had an inhibitory effect on phenylacetaldehyde in a specific concentration range. The correlation analysis of the effects of furfural and 5-methyl-2-furfural on phenylacetaldehyde and PhIP showed that there were strong positive correlations between them (Pearson’s r = 0.8934, R^2^ = 0.76933; Pearson’s r = 0.84221, R^2^ = 0.65117) (Figure S2). They suggested that the reduction of PhIP formation by furfural (5-methyl-2-furfural) is related to the decrease in phenylacetaldehyde.

The reason for the decrease in phenylacetaldehyde in the model may be the decrease in phenylacetaldehyde formed by Phe conversion, or it could be due to the consumption of phenylacetaldehyde as it reacts with furfural (5-methyl-2-furfural). To determine the cause, we studied the reaction model of furfural (5-methyl-2-furfural) with phenylacetaldehyde. As seen from Figure 2e,f, furfural (5-methyl-2-furfural) had no significant effect on phenylacetaldehyde, indicating that they do not directly react with phenylacetaldehyde. The above information further indicated that furfural (5-methyl-2-furfural) may reduce the formation of PhIP by reducing phenylacetaldehyde converted from Phe.

**Figure 3:**
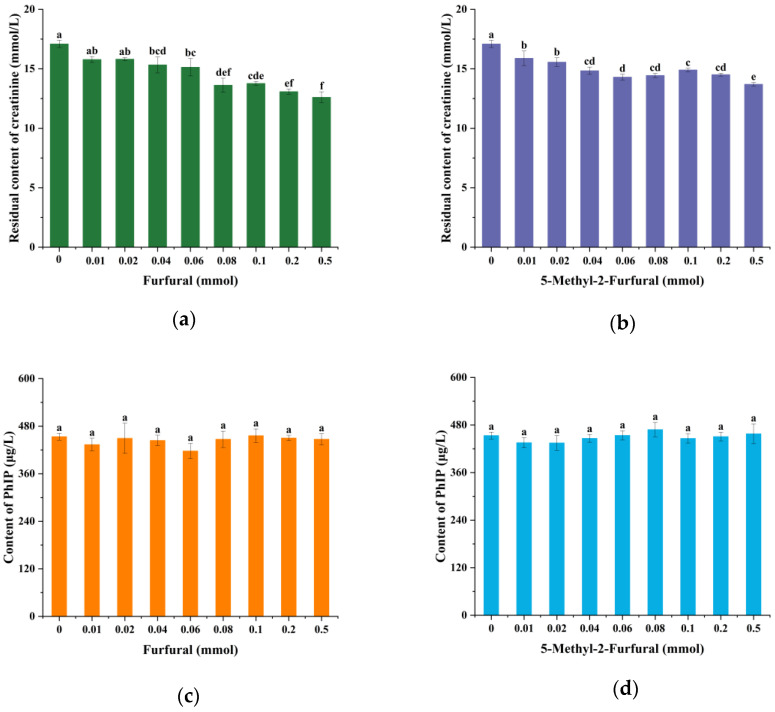
The change in amount of creatinine in the reaction models of furfural (5-methyl-2-furfural) with creatinine (**a**,**b**); change in amount of PhIP in the reaction models of furfural (5-methyl-2-furfural) with PhIP (**c**,**d**). Different lowercase letters indicate significant difference (*p* < 0.05).

In addition, whether the reaction of furfural (5-methyl-2-furfural) with creatinine affects the formation of PhIP through creatinine was studied. As shown in Figure 3a,b, the data showed that adding different amounts of furfural (5-methyl-2-furfural) had no significant effect on creatinine (*p* < 0.05). These results indicated that furfural (5-methyl-2-furfural) does not react significantly with creatinine.

In addition, to further explore whether furfural (5-methyl-2-furfural) affects the reduction of produced PhIP, we reacted different concentrations of furfural (5-methyl-2-furfural) with PhIP. Figure 3c,d show that there was no significant change in the residual amount of PhIP after the reaction (*p* < 0.05), indicating that the effects of furfural and 5-methyl-2-furfural on PhIP were not achieved by direct consumption of the PhIP generated.

From the above results, it can be concluded that the reason furfural (and 5-methyl-2-furfural) reduces PhIP is that it does not react with creatinine, phenylacetaldehyde, or PhIP but only acts in the conversion stage of Phe to phenylacetaldehyde. It can be seen that the mechanism of this result may be different from the mechanism in which some compounds (such as flavonoids, anthocyanins, and rutin mangiferin) can inhibit the formation of PhIP by binding to phenylacetaldehyde [33,34,35,36,37,38]. Mechanisms that inhibit the formation of heterocyclic amines, such as polyphenols [39] and catechins [40], act through either antioxidant activity or free radical scavenging capacity. It should be noted that furfural and 5-methyl-2-furfural cannot be used as food additives, but they are significant in revealing the mechanism of glucose affecting PhIP formation. Therefore, the reaction pathway and underlying mechanism of furfural and 5-methyl-2-furfural affecting Phe in the process of PhIP formation need to be further studied.

### 2.3. Identification of Reaction Products in Chemical Models

**Figure 4:**
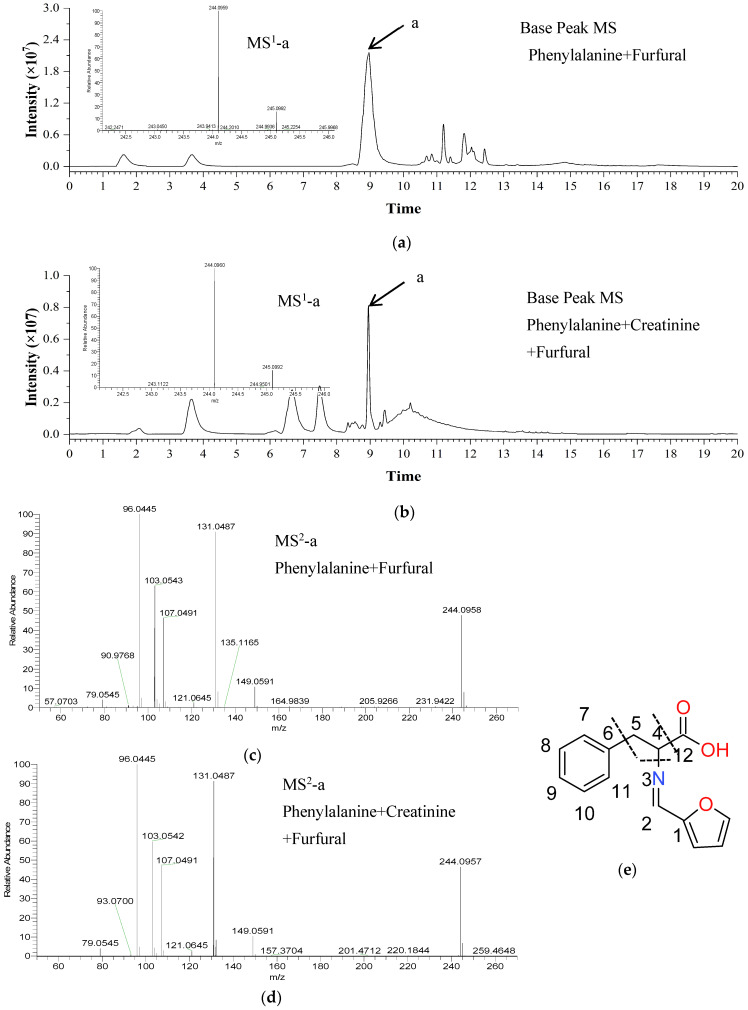
Identification of reaction products by UHLC-HRMS. The total ion chromatogram (TIC) of furfural/phenylalanine (Phe) and furfural/Phe/creatinine reaction products and the MS^1^ spectra of compound a (**a**,**b**). The corresponding MS^2^ spectra (**c**,**d**) of compound a. The possible molecular structure of compound a (**e**).

The effects of furfural and 5-methyl-2-furfural on Phe during the formation of PhIP were studied by different reaction products of furfural (5-methyl-2-furfural)/Phe, furfural (5-methyl-2-furfural)/Phe/creatinine, and the control group. The conditions for UPLC-HRMS were optimized according to the reaction model samples. The total ion chromatogram (TIC) for samples was acquired using full-scan analysis in positive ion mode. As shown in Figure 4a,b, in the TIC of the furfural/Phe and furfural/Phe/creatinine reaction samples, it was observed that there was a new peak (compound a) at the retention time of 9.03 min in both samples: the MS^1^ spectrum showed [M + H]^+^ at *m*/*z* 244.0960. The elemental composition was set to C, H, O, and N, and the allowable deviation was not more than five ppm. The predicted molecular formula was C_14_H_13_NO_3_, with a calculated mass relative error of 3.2 × 10^−6^.

Based on the structure of the reactant, the structural formula of the product compound a was deduced by analyzing the MS^2^ spectrum characteristic ions peak resulting from the dissociation of the molecule ion peak at *m*/*z* 244.0960 ([M + H]^+^), which produced characteristic fragment ion peaks at *m*/*z* 149.0591, 131.0485, 107.0491, 103.0543, 96.0445, and 79.0545 (Figure 4c,d). The MS^2^ spectrum yielded a characteristic peak at *m*/*z* 79.0545, suggesting the presence of a benzene ring in the parent ion structure. The MS^2^ spectrum yielded a characteristic peak at *m*/*z* 107.0491, which could be analyzed as [C_8_H_10_ + H]^+^, indicating that the parent ion structure contains ethylbenzene. The MS^2^ spectrum yielded a characteristic peak at *m*/*z* 96.0445, which could be analyzed as C_5_H_6_NO^+^, which suggests the presence of an oxygen-containing five-membered heterocycle in the parent ion structure. The MS^2^ spectrum yielded a characteristic peak at *m*/*z* 131.0485, which could be analyzed as C_9_H_9_N_2_^+^, which suggests that the parent ion structure lost a COOH^−^ and a furan (C_4_H_4_O). The MS^2^ spectrum yielded a characteristic peak at *m*/*z* 149.0591, which could be analyzed as [C_9_H_11_NO + H]^+^, which suggests that the parent ion structure lost an OH^−^ and a benzene ring (C_6_H_6_). The fragment ions mentioned above indicate the presence of a benzene ring, a five-membered heterocyclic ring, and a carboxylic group within the core structure, with a degree of unsaturation of 8. Additionally, one degree of unsaturation in the structure suggests that the remaining part consists of a condensation bond formed through the condensation of a carbonyl group with an amine. In summary, the possible structural formula of compound a was shown in Figure 4e, and the compound was speculated to be 2-((furan-2-ylmethylene) amino)-3-phenylpropanoic acid.

**Figure 5:**
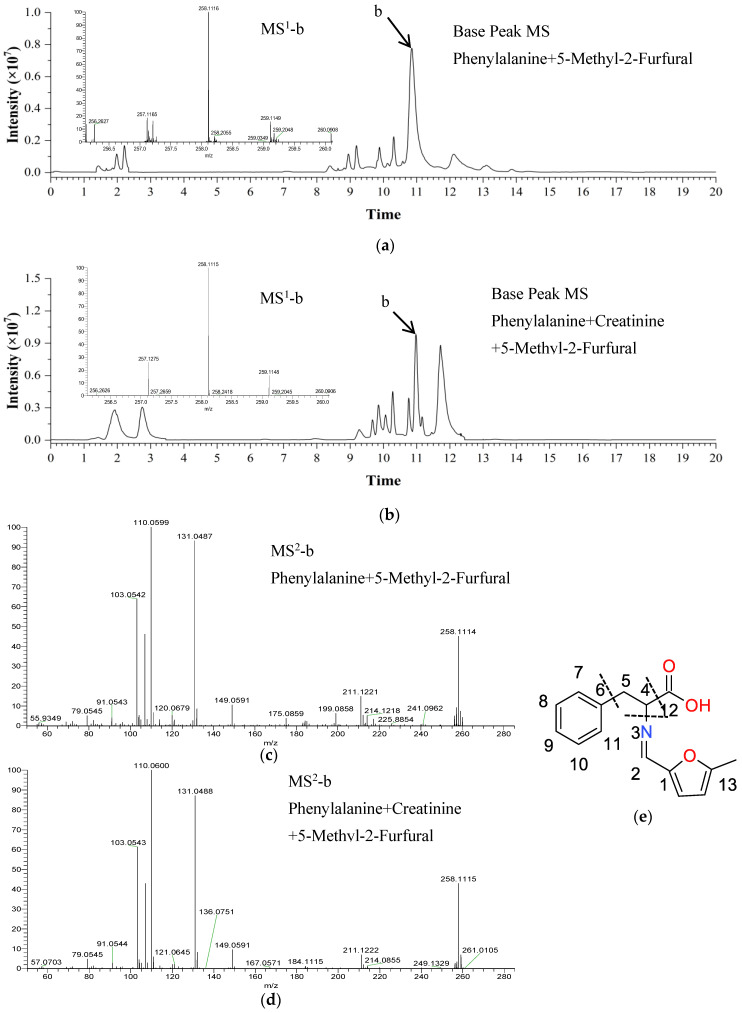
Identification of reaction products by UHLC-HRMS. The total ion chromatogram (TIC) of 5-methyl-2-furfural/phenylalanine (Phe) and 5-methyl-2-furfural/Phe/creatinine reaction products, and the MS^1^ spectra of compound b (**a**,**b**). The corresponding MS^2^ spectra (**c**,**d**) of compound b. The possible molecular structure of compound b (**e**).

In addition, a new peak (compound b) was observed in the TIC of the 5-methyl-2-furfural/Phe/creatinine and 5-methyl-2-furfural/Phe reaction samples when the retention time was 10.99 min: the MS^1^ spectrum showed [M + H]^+^ at *m*/*z* 258.1115 (Figure 5a,b). The predicted molecular formula was C_15_H_15_NO_3_, with a calculated mass relative error of 3.8 × 10^−6^. The fragmentation of molecular ions produced the MS^2^ spectrum characteristic peaks at *m*/*z* 211.1222, 149.0591, 110.0600, 103.0543, and 79.0545 (Figure 5c,d). The compound b MS^2^ spectrum peaks at *m*/*z* 79.0545, 103.0543, and 149.0591 were the same as compound a, indicating that compound b and compound a had partially the same structure. The difference is that compound b has a fragment ion peak at *m*/*z* 110.0600, and compound a has a fragment ion peak at *m*/*z* 96.0445, with a CH_2_ difference between them. It was speculated that the structure of compound b was one more CH_2_ than the compound a structure. In addition, their degree of unsaturation is the same. It was speculated that compound b may be 2-(((5-methylfuran-2-yl)methylene)amino)-3-phenylpropanoic acid, and the structural formula is shown in Figure 5e.

### 2.4. Reaction Pathways of Furfural and 5-Methyl-2-Furfural Affecting the Formation of PhIP

**Figure 6:**
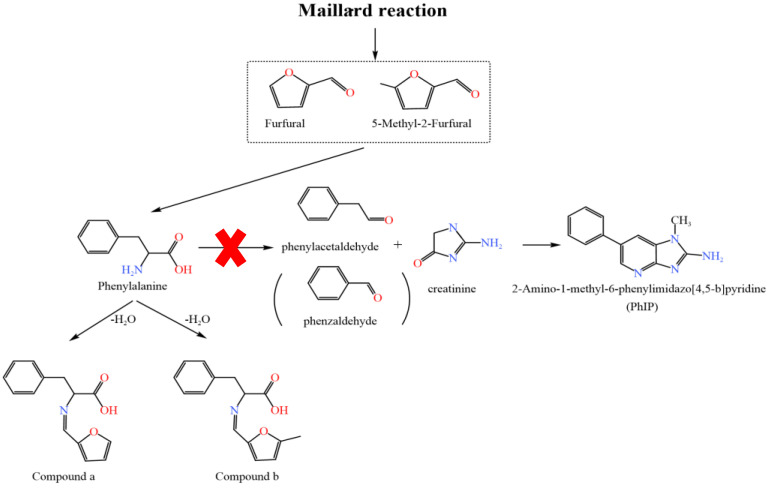
Proposed pathway for furfural and 5-methyl-2-fural inhibiting PhIP formation in the chemical models.

Based on the above results and the formation mechanism of PhIP [41,42], the reaction pathways for the effect of furfural and 5-methyl-2-furfural on the mitigation of PhIP in the model systems are summarized in Figure 6. The main pathway is as follows: The amino group of Phe preferentially undergoes a condensation reaction with the carbonyl group of furfural (5-methyl-2-furfural), leading to the formation of a Schiff base upon dehydration. This reaction pathway effectively blocks the decarboxylation and deamination of Phe to form phenylacetaldehyde (and benzaldehyde), thereby reducing the formation of PhIP. The primary reason for this reduction is that the condensation reaction is thermodynamically more favorable than the decarboxylation and deamination reactions of Phe. Interestingly, both creatinine and PhIP contain amino groups, yet they do not react with furfural (5-methyl-2-furfural). The rationale behind this lies in the fact that the nitrogen atoms in the amino groups of creatinine and PhIP are connected to the carbon–carbon double bond to form a conjugated structure, which leads to the weakening of the nucleophilicity of the amino group and is not easy to react. In contrast, The carbonyl group of furfural/5-methylfurfural undergoes a condensation reaction with the amino group of phenylalanine, resulting in the loss of one water molecule and the formation of a Schiff base [43]. It is evident that furfural and 5-methyl-2-furfural affect PhIP formation by promoting the conversion of Phe to Schiff bases, thereby reducing the formation of the intermediate phenylacetaldehyde (and benzaldehyde) in the PhIP formation. Interestingly, some researchers have found that acrolein, a lipid oxidation product, can also affect PhIP formation by forming a Schiff base with Phe [10]. This suggests that aldehydes can influence Phe’s subsequent reaction through the ammonia condensation reaction.

### 2.5. Verification of the Reaction Pathway in Roasted Pork Patties

**Figure 7:**
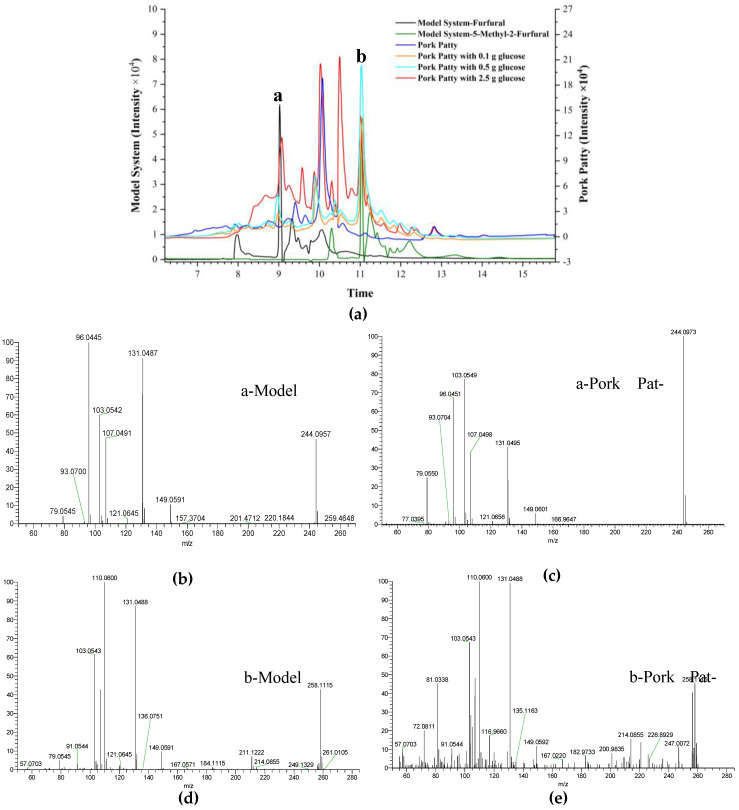
The chromatograms of compounds a and b in the chemical models and pork patties (**a**). The MS^2^ spectra of compound a in the chemical models (**b**) and roasted pork patties with glucose (**c**). The MS^2^ of compound b in chemical models (**d**) and roasted pork patties with glucose (**e**).

In food systems, glucose produces furfural and 5-methyl-2-furfural through the Maillard reaction. In order to verify that our speculated furfural and 5-methyl-2-furfural inhibition of the PhIP formation pathway exists in real food systems, we directly added furfural and 5-methyl-2-furfural to pork patties and detected the PhIP content. The data clearly show that furfural and 5-methyl-2-furfural significantly reduced the formation of PhIP in pork patties (Figure S3). Despite the addition of furfural and 5-methyl-2-furfural, PhIP was still detected in the experiment. This occurrence is attributed to the complex nature of the Maillard reaction in real food systems. Our study shows that while furfural and 5-methyl-2-furfural can inhibit the formation of PhIP in controlled model systems, other precursors and reaction pathways present in actual food may influence the overall outcome. Furthermore, we isolated and identified the substances in the roasted pork patties with glucose. To verify the presence of the proposed pathway by which furfural and 5-methyl-2-furfural inhibit the formation of PhIP in real food systems, we performed isolation and identification of the compounds in roasted pork patties with glucose. Excitingly, we discovered compounds a and b in the pork patties with glucose (Figure 7); the relative amount of compounds a and b were changed by adding low, medium, and high levels of glucose. The presence of compounds a and b in pork patties without glucose was not obvious, from which we can deduce that glucose can indeed affect PhIP formation by producing furfural and 5-methyl-2-furfural. Considering these results, we deduced that glucose produces furfural and 5-methyl-2-furfural through the Maillard reaction, which then react with Phe to inhibit the formation of PhIP.

Furthermore, this indicates that another pathway exists by which glucose inhibits the formation of PhIP, thereby providing additional evidence to support our previous research that proposed that the pathways by which reducing sugars inhibit the formation of PhIP in meat are not unique [30].

## 3. Materials and Methods

### 3.1. Chemicals and Materials

The standard of 2-Amino-1-methyl-6-phenylimidazo[4,5-b]pyridine (PhIP) standard was purchased from Shanghai Aladdin Biochemical Technology Co., Ltd. (Shanghai, China). Creatinine, phenylalanine (Phe), phenylacetaldehyde, and ammonium formate were obtained from Sigma-Aldrich (Shanghai) Trading Co., Ltd. (Shanghai, China). Furfural and 5-methyl-2-furfural were supplied by ANPEL-TRACE Standard Technical Services (Shanghai) Co., Ltd. (Shanghai, China). MS-grade methanol, acetonitrile, ammonium acetate, and formic acid were obtained from Thermo Fisher Scientific (Waltham, MA, USA). Pure water was purchased from Wahaha Jinan Industrial Park (Jinan, China).

### 3.2. Model Systems

Glucose/amino acids chemical models: These were based on the different proportions of the main amino acids (Ala:Arg:Gln:Glu:Gly:Cys:Lys:Phe:Tyr = 6:1:1.8:1.5:1.7:2:1.7:1:1.7) in Duroc pork mixed with glucose (1–500 μmol) and dissolved in 5 mL diethylene glycol (86%)/water (14%) (pH = 6.4) in screw-cap test vials, and the vials were heated at 180 °C for 1 h and cooled in ice water. All experiments were repeated three times.

Phe/creatinine/furfural (5-methyl-2-furfural) chemical models: Phe (0.1 mmol) and creatinine (0.1 mmol) with different amounts of furfural (5-methyl-2-furfural) (0.01–0.50 mmol) were weighed and dissolved in 5 mL of diethylene glycol (86%)/water (14%) (pH = 6.4) in screw-cap test vials. The vials were heated at 180 °C for 1 h and then cooled in ice water. All experiments were repeated three times.

Phe/furfural (5-methyl-2-furfural), creatinine/furfural (5-methyl-2-furfural), phenylacetaldehyde/furfural (5-methyl-2-furfural), and PhIP/furfural (5-methyl-2-furfural) model systems: Phe (0.1 mmol), creatinine (0.1 mmol), phenylacetaldehyde (10 μmol), or PhIP (10 nmol) were each reacted with different amounts of furfural (5-methyl-2-furfural) and were weighed and dissolved in 5 mL of diethylene glycol (86%)/water (14%) (pH = 6.4) in screw-cap test vials. The vials were heated at 180 °C for 1 h and then cooled in ice water. All experiments were repeated three times.

Roast Pork Patties: Duroc pork was obtained from a local supermarket in Jinan, China. furfural (5-methyl-2-furfural) (2.5–25 μL) or glucose (0.5–2.5 g) was added to 50 g of minced pork (pH = 6.1), respectively. The mixture was shaped with a specific mold (thickness 5 mm) and then roasted at 180 °C for 30 min.

### 3.3. HPLC Analysis of Furfural and 5-Methyl-2-furfural

The sample was diluted 20-fold with acetonitrile, then centrifuged and filtered by an organic membrane (0.22 μm). The HPLC instrument (LC20-AT, Shimadzu, Kyoto, Japan) was equipped with a UV detector, a column oven, and an automatic sampler. The samples were separated by Kromasil C18 column (250 mm × 4.6 mm, 5 μm). The injection volume was 20 μL, the flow rate was 1.0 mL/min, the column temperature was 40 °C, the detection wavelength was 365 nm, and the mobile phase was acetonitrile/water (70:30, *v*/*v*).

### 3.4. LC-MS/MS Analysis of PhIP

For the chemical model, 2 mL of sample was added to the Cleanert S C18 column, which had been activated with methanol. This was followed by elution with 10 mL of deionized water, flushing with 2 mL of methanol and a mixture of methanol and ammonia (19:1, *v*/*v*), collection of the elution liquid, drying of the elution liquid with nitrogen, reconstitution, and dilution with 10 times the volume of methanol; the sample was then filtered by an organic membrane (0.22 μm) and subjected to LC-MS/MS analysis.

For the pork patties, the previously reported method [33,34] was modified. The lyophilized sample (6.0 g) was weighed, and 40 mL of 1 mol/L NaOH was added for homogenization for 2 min. It was then mixed well with 15 g of diatomaceous earth, ultrasonicated with 50 mL of ethyl acetate for 10 min, and extracted for 20 min, and the process was repeated twice. The ethyl acetate layer was collected, and low-temperature rotary evaporation was performed. The concentrated solution was transferred to a Cleanert S C18 column and washed with 10 mL of deionized water. It was flushed with 2 mL of methanol and a mixture of methanol and ammonia (19:1, *v*/*v*) and evaporated under nitrogen. The residue was dissolved in 500 μL of methanol and filtered through an organic membrane (0.22 μm) for LC-MS/MS analysis.

The method for detecting PhIP followed our previous study [32]. Samples were analyzed using an Agilent 1200 Series LC system coupled to an Agilent 6410 triple quadrupole mass spectrometer (Agilent, Waldbronn, Germany) in positive electrospray ionization (ESI+) mode. The column was a Zorbax Eclipse Plus C18 (2.1 × 150 nm, 3.5 μm), and the detection mode was multiple reaction detection (MRM). The mass spectral transition pattern of PhIP is *m*/*z* 225.3 → *m*/*z* 210.

### 3.5. HPLC Analysis of Creatinine

The detection method for creatinine was adjusted based on our previous study [32]. The sample was diluted twenty times with ultrapure water before HPLC (LC20-AT, Shimadzu, Japan) analysis. A Kromasil C18 reversed-phase column (250 mm × 4.6 mm, 5 μm) was used for the separation, and the mobile phases were 25 mM KH_2_PO_4_ buffer (phase A, pH = 6.9) and acetonitrile (phase B). The isocratic elution was 10% B for 10 min with a flow rate of 0.8 mL/min. The detection wavelength was 215 nm, and the injection volume was 20 μL.

### 3.6. HPLC Analysis of Phe

The Phe was analyzed according to the kit instructions provided by the manufacturer. After heating and cooling, the reaction solution was diluted with ultrapure water. Two derivative reagents, A and B, were diluted with diluent to one-fifth of the original concentration. The sample solution was accurately placed in a test tube, and diluted solutions A and B were added. The mixture was shaken well and reacted at room temperature for 60 min. Then, n-hexane was added to dilute the solution twice, and it was passed through an organic filter membrane (0.22 μm) for equipment analysis. All the experiments were performed in triplicate.

The detection of Phe was performed according to the operating instructions of the purchased matching reagent kit. The HPLC was an LC20-AT (Shimadzu, Japan) equipped with a UV detector, column oven, and autosampler. And an Ultimate Amino Acid column (4.6 × 250 mm, 5 μm) was used for the separation.

### 3.7. GC-MS Analysis of Phenylacetaldehyde

After heating and cooling the reaction mixture, 3 mL of the reaction solution was transferred to a plugged test tube. Then, 4 mL of the mixture of ethyl acetate and n-hexane (*v*:*v* = 3:1) was added, and the mixture was vortexed for 3 min (repeat three times). The separated organic layer was removed by rotary evaporation at low temperature, and then the residue was redissolved in 3 mL ethyl acetate, followed by filtering with a 0.22 μm organic filter membrane, and finally analyzed by GC-MS.

The determination of phenylacetaldehyde was performed according to the GC-MS method of the previous literature with minor modifications [35]. Phenylacetaldehyde was analyzed on a Thermo Fisher Scientific TRACE 1300 gas chromatograph coupled with a TSQ 8000 EVO triple quadrupole mass spectrometer (Thermo Fisher Scientific, Waltham, MA, USA) and a Thermo Fisher Scientific TG-5MS column (30 m × 0.25 mm × 0.25 μm). The injection amount was 1 μL in splitless mode. The column’s program of temperature rise was as follows: 40 °C (2 min), 40–230 °C at 8 °C/min, and 230 °C (5 min). The MS transfer line temperature was held at 250 °C, and the ion source temperature was held at 210 °C. The carrier gas was helium and carrier flow was 1 mL/min; split flow was 10 mL/min. The identification and quantification were performed by comparing the retention time of phenylacetaldehyde with standards using an external standard method. Specifically, a calibration curve (R^2^ = 0.9993) was established with gradient concentrations of phenylacetaldehyde standard (2–100 μmol/L). LOD in samples was 0.8 pmol/mL, and LOQ in samples was 2.7 pmol/mL Sample concentrations were calculated via linear regression equation.

### 3.8. UPLC-HRMS Separation and Identification of Reactants

Phe/creatinine/furfural (5-methyl-2-furfural) model systems: To identify the characterized compounds in the model system, the reacted mixtures of the Phe/creatinine/furfural (5-methyl-2-furfural) and Phe/furfural (5-methyl-2-furfural) and creatinine/furfural (5-methyl-2-furfural) model systems were diluted with MeOH to 1:10, filtered by an organic membrane (0.22 μm), and analyzed by UPLC-HRMS (Thermo Fisher Scientific UltiMate 3000 Ultra HPLC with Q Exactive HF mass spectrometer utilizing positive electrospray ionization (ESI+)).

Pork patties: The patties were ground to a powder using an IKA a11 analytical grinder (Janke & Kunkel, Staufen, Germany). An amount of 5 g of patty powder was mixed with and 25 mL of ultrapure water (0.1% formic acid) and then homogenized for 2 min. Then, 50 mL of ethyl acetate was extracted twice and centrifuged at 10,000× *g* 4 °C for 10 min using a High-Speed Refrigerated Centrifuge CR21N (Hitachi, Tokyo, Japan). The supernatant was collected, and the solution was rotated and evaporated. The mixture was dissolved in 1 mL of methanol and filtered by an organic membrane (0.22 μm) for UPLC-HRMS analysis.

The detection of unknown objects was appropriately modified based on the previous literature [36]. Unknown object determination was performed on a Thermo Fisher Scientific UltiMate 3000 Ultra HPLC with Q Exactive HF mass spectrometer utilizing positive electrospray ionization (ESI+). A Hypersi GOLD Liquid chromatography column (100 × 2.1 mm, 3 μm) was employed to separate unknown objects at a temperature of 35 °C; 100% acetonitrile (B) and 0.5% formic acid (A) were used as mobile phases, and the injection volume was 5 μL at a flow rate of 0.2 mL/min. The gradient elution program was as follows: 0–1.5 min, 1% B; 1.5–14 min, 99% B; 14–14 min, 99–1% B; 14–20 min, 1% B. The scanning mode of mass spectrometry detection was Full MS-ddMS^2^, HESI source condition: sheath gas flow rate was 35, Aux gas flow rate was 10, spray voltage was 3.50 kV, capillary temperature was 350 °C, S-lens RF level was 50.0, and aux gas heater temperature was 300 °C. Data were compared and analyzed between the control groups and the sample groups using Xcalibur 4.3 to identify potential reactants and reaction pathways.

### 3.9. Statistical Analysis

All experiments were conducted with three independent sample replicates (n = 3), and each sample was tested in triplicate. The levels of PhIP, its precursor substances, and related intermediates were expressed as the mean ± standard deviation (SD) of three parallel experiments. ANOVA and Duncan’s test (IBM SPSS Statistics 21) were used to compare the significant differences among the treatments, and the significance between the sample means was designated as a probability (*p*) value < 0.05. Origin 2021 was used to draw all the figures, and ChemDraw 2022 64-bit was used to map the reaction pathway.

## 4. Conclusions

In conclusion, this study investigated the effects of furfural and 5-methyl-2-furfural on the formation of PhIP in both chemical models and roasted pork patties. Our findings demonstrate that furfural and 5-methyl-2-furfural showed an inhibitory effect against PhIP formation in chemical models. The interaction between furan compounds (furfural and 5-methyl-2-furfural) and Phe rather than between furan compounds and creatinine and PhIP itself played an important role in reducing PhIP formation in chemical model systems. Further mechanism studies revealed that furfural and 5-methyl-2-furfural individually capture Phe to form corresponding Schiff bases (compounds a and b). This reaction reduces the formation of intermediate phenylacetaldehyde (or benzaldehyde), blocks the PhIP formation pathway, and decreases PhIP content. The above pathway was finally confirmed in the roasted pork patties with glucose, further demonstrating that the effect of glucose on PhIP was not only through α-dicarbonyl compounds but also through the above pathway. Given the complexity of the Maillard reaction and the presence of multiple amino acids in food systems, further research is warranted to explore the impact of other Maillard reaction products on the formation of PhIP. This will help in understanding the comprehensive effects of redusscing sugars and other amino acids on the formation of heterocyclic amines. The conclusions of this study still require further research on the relationships between furfural (5-methyl-2-furfural) produced by glucose and various aspects, such as meat processing technology, sensory evaluation, nutrition, and safety, before they can be applied in practice.

## Data Availability

Data are available upon reasonable request.

## Supplementary Materials

The following supporting information can be downloaded at: https://www.mdpi.com/article/10.3390/molecules30061254/s1, Table S1: The amount of PhIP generation (μg/L) and inhibition rate (%). Figure S1: (a) The mount of furfural (F) formed by adding different amounts of glucose to the glucose/amino acids model; (b) The mount of 5-methyl-2-furfural (5-MF) formed by adding different amounts of glucose to the glucose/amino acids model. Different lowercase letters indicate significant different (*p* < 0.05); Figure S2: (a) Correlation analysis between the effect of furfural (F) on PhIP and the effect on phenylacetaldehyde production; (b) Correlation analysis between the effect of 5-methyl-2-furfural (5-MF) on PhIP and the effect on phenylacetaldehyde production; Figure S3. (a) Effects of furfural (F) on the formation of PhIP in the pork patties; (b) Effects of 5-methyl-2-fural (5-MF) (b) on the formation of PhIP in the pork patties. Different lowercase letters indicate significant different (*p* < 0.05).
