# Supplementary material for: Role of Furfural and 5-Methyl-2-furfural in Glucose-Induced Inhibition of 2-Amino-1-methyl-6-phenylimidazo[4,5-b]pyridine (PhIP) Formation in Chemical Models and Pork Patties
Source: Molecules. 2025 Mar 11;30(6):1254. doi: 10.3390/molecules30061254 (PMC11944780; doi:10.3390/molecules30061254)
Supplement: Supplementary file 1 [file molecules-30-01254-s001.zip › molecules-3397130-supplementary.pdf]

# Role of Furfural and 5-Methyl-2-furfural in Glucose-Induced Inhibition of 2-Amino-1-methyl-6-phenylimidazo[4,5-b]pyridine (PhIP) Formation in Chemical Models and Pork Patties

**Yuexia Qin [1], Zhuyu Zheng [1], Di Liu [1], Shuhua Sun [2], Xiaolei Zhao [1], Lei Lv [1], Dengyu Xie [2], Zhonghui Han [1,2,*] and Jinxing He [1,*]**

[1] Shandong Key Laboratory of Healthy Food Resources Exploration and Creation, College of Food Science and Engineering, Qilu University of Technology (Shandong Academy of Sciences), Jinan 250353, China; 10431221122@stu.qlu.edu.cn (Y.Q.); 202395093001@stu.qlu.edu.cn (Z.Z.); liudi92304@163.com (D.L.); zhaoxiaolei@qlu.edu.cn (X.Z.); lvlei831005@qlu.edu.cn (L.L.)

[2] Shandong Engineering Research Center of Food Nutrition and Active Health, Binzhou Key Laboratory of Corn Deep Processing Technology, Shandong Xiwang Foodstuffs Co., Ltd., Binzhou 256200, China; sunshuhua7677@126.com (S.S.); dengnet@126.com (D.X.)

* Correspondence: zhhan@qlu.edu.cn (Z.H.); jinhe@qlu.edu.cn (J.H.)

**Table S1.** The amount of PhIP generation (μg/L) and inhibition rate (%)

| Compound name | The amount of furans added （mmol） | 0 | 0.01 | 0.02 | 0.04 | 0.06 | 0.08 | 0.10 | 0.20 | 0.50 |
|---|---|---|---|---|---|---|---|---|---|---|
| furfural | Amount of PhIP (μg/L) | 1319.41±103.41$^{a}$ | 1254.66±22.14$^{b}$ | 1155.67±114.97$^{b}$ | 868.99±78.67$^{c}$ | 904.32±99.40$^{c}$ | 838.78±119.48$^{c}$ | 238.33±19.98$^{d}$ | 300.30±48.70$^{d}$ | 253.50±75.93$^{d}$ |
| | Inhibition rate (%) | 0 | 4.91 | 12.41 | 34.13 | 31.46 | 36.43 | 81.94 | 77.24 | 80.79 |
| 5-methyl-2-furfural | Amount of PhIP (μg/L) | 1319.41±103.41$^{b}$ | 1732.65±5.98$^{a}$ | 1332.26±29.55$^{b}$ | 1172.41±21.48$^{c}$ | 1085.01±24.49$^{d}$ | 1046.44±94.94$^{d}$ | 705.10±23.67$^{e}$ | 642.52±32.07$^{e}$ | 446.95±9.70$^{f}$ |
| | Inhibition rate (%) | | -31.32 | -0.97 | 10.76 | 17.77 | 20.69 | 45.56 | 51.30 | 66.13 |

* Different superscript letters in the same column represent a significant difference ($P < 0.05$).

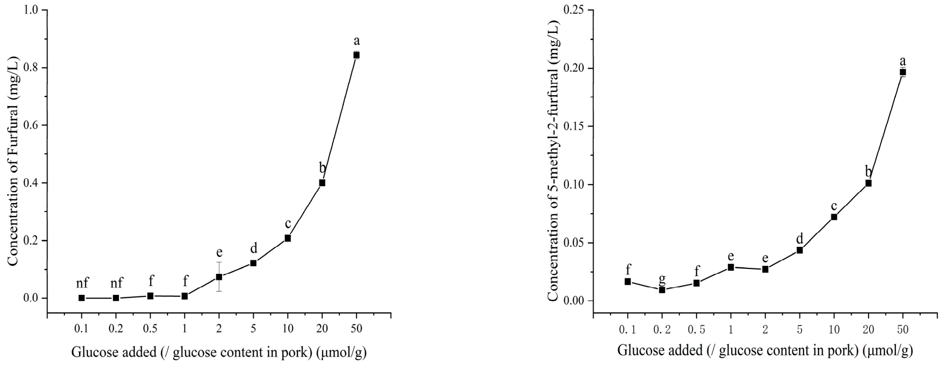


(**a**) (**b**)

**Figure S1.** (a) The mount of furfural (F) formed by adding different amounts of glucose to the glucose/amino acids model; (b) The mount of 5-methyl-2-furfural (5-MF) formed by adding different amounts of glucose to the glucose/amino acids model. Different lowercase letters indicate significant different ($P$ < 0.05)

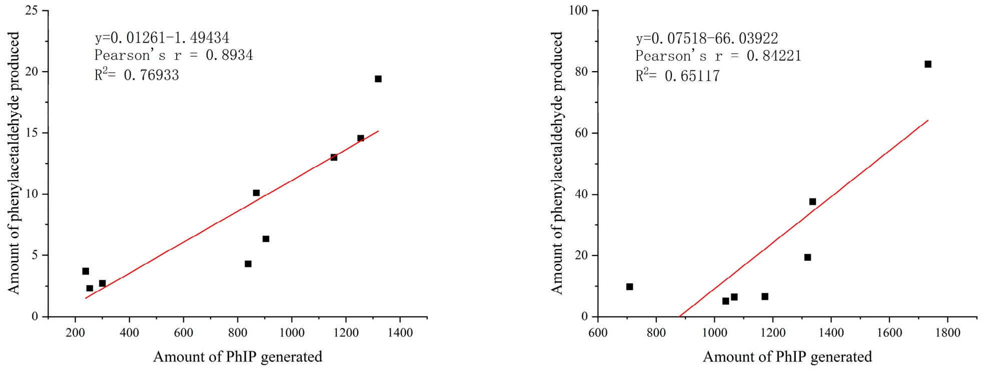


(**a**) (**b**)

**Figure S2.** (a) Correlation analysis between the effect of furfural (F) on PhIP and the effect on phenylacetaldehyde production; (b) Correlation analysis between the effect of 5-methyl-2-furfural (5-MF) on PhIP and the effect on phenylacetaldehyde production.

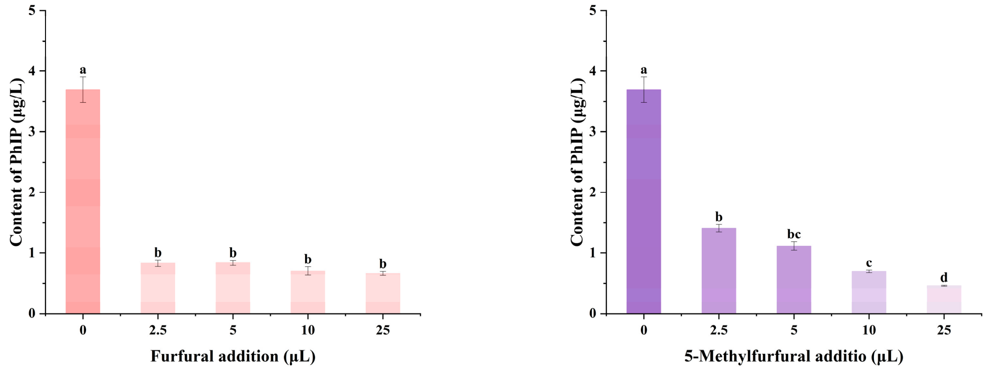


(**a**) (**b**)

**Figure S3.** (a) Effects of furfural (F) on the formation of PhIP in the pork patties; (b) Effects of 5-methyl-2-fural (5-MF) (b) on the formation of PhIP in the pork patties. Different lowercase letters indicate significant different ($P < 0.05$).
